# The impact of metabolic stress on anti-tumor immunity in laboratory mice

**DOI:** 10.1186/2051-1426-1-S1-P122

**Published:** 2013-11-07

**Authors:** Kathleen M Kokolus, Maegan L Capitano, Jason W Eng, Bonnie L Hylander, Christopher J Gordon, Scott I Abrams, Elizabeth A Repasky

**Affiliations:** 1 Department of Immunology, Roswell Park Cancer Institute, Buffalo, NY, USA; 2 US Environmental Protection Agency, Research Triangle, NC, USA

## 

There is an underappreciated amount of physiological stress placed on laboratory mice including that due to standard, required housing at sub-thermoneutral temperature. This condition results in an increase in metabolism needed to produce heat necessary to maintain body temperature. Since body temperature maintenance is a high priority for survival, we hypothesized that metabolic energy may be allocated to body temperature maintenance at the expense of the anti-tumor immune response under standard cool housing temperatures. At ambient temperatures of 30-31°C, which is considered the thermoneutral temperature (TT) for mice, resting metabolic rate is sufficient to generate enough heat to maintain body temperature. However, mice are kept at a cooler standard temperature (ST; 21-23°C). Our data reveals increased heat-seeking behavior in tumor-bearing mice housed at ST indicating a physiological drive for additional ambient warmth, likely needed to lessen the energetic burden of maintaining body temperature. We found significantly delayed tumor growth rates and a reduction in metastasis in mice housed at TT compared to those at ST. We investigated immunodeficient mice and mice depleted for CD8+ T cells and found no differences in tumor growth between mice housed at ST and TT suggesting a role for the T cell dependent immune response. Because the activation of CD8+ T cells is an energetically demanding process we predicted the function of these cells would be diminished in animals at ST since more metabolic resources may be diverted to heat production. According to this hypothesis we saw an increase in glucose uptake in the lymph nodes of tumor-bearing mice as well as an increase in Glut-1 expression on splenic CD8+ T cells in mice maintained at TT suggesting increased metabolic activity in these cell populations. Our studies also revealed more antigen specific CD8+ T cells within the lymph node and tumor microenvironment of animals housed at TT compared to those housed at ST. Finally, our data has also shown a significantly increased number of immunosuppressive cell subsets including Gr-1+CD11b+ myeloid derived suppressor cells and Foxp3+ cells in mice maintained at ST compared to those at TT. These data demonstrate that tumor growth and anti-tumor immune control in laboratory mice is highly influenced by cold stress. We hypothesize that the amount of energy available to T cells under standard cool housing conditions is reduced. Since metabolism and its role in T cell activation is increasingly modeled in laboratory mice, it is essential to take into account the impact that housing temperature has on anti-tumor immune activity.

